# Determination of Mean Intrinsic Flexural Strength and Coupling Factor of Natural Fiber Reinforcement in Polylactic Acid Biocomposites

**DOI:** 10.3390/polym11111736

**Published:** 2019-10-23

**Authors:** Quim Tarrés, Helena Oliver-Ortega, F. Xavier Espinach, Pere Mutjé, Marc Delgado-Aguilar, José A. Méndez

**Affiliations:** 1 LEPAMAP Group, Department of Chemical Engineering, University of Girona, 17003 Girona, Spain; helena.oliver@udg.edu (H.O.-O.); pere.mutje@udg.edu (P.M.); jalberto.mendez@udg.edu (J.A.M.); 2 Càtedra de Processos Industrials Sostenibles, University of Girona, 17003 Girona, Spain; 3 Design, Development and Product Innovation, Dept. of Organization, Business, University of Girona, 17003 Girona, Spain; francisco.espinach@udg.edu

**Keywords:** PLA composites, bleached softwood fibers, bio-based materials, biodegradable materials, micromechanics

## Abstract

This paper is focused on the flexural properties of bleached kraft softwood fibers, bio-based, biodegradable, and a globally available reinforcement commonly used in papermaking, of reinforced polylactic acid (PLA) composites. The matrix, polylactic acid, is also a bio-based and biodegradable polymer. Flexural properties of composites incorporating percentages of reinforcement ranging from 15 to 30 wt % were measured and discussed. Another objective was to evaluate the strength of the interface between the matrix and the reinforcements, using the rule of mixtures to determine the coupling factor. Nonetheless, this rule of mixtures presents two unknowns, the coupling factor and the intrinsic flexural strength of the reinforcement. Hence, applying a ratio between the tensile and flexural intrinsic strengths and a defined fiber tensile and flexural strength factors, derived from the rule of mixtures is proposed. The literature lacks a precise evaluation of the intrinsic tensile strength of the reinforcements. In order to obtain such intrinsic tensile strength, we used the Kelly and Tyson modified equation as well as the solution provided by Bowyer and Bader. Finally, we were able to characterize the intrinsic flexural strengths of the fibers when used as reinforcement of polylactic acid.

## 1. Introduction

In the last decade, terms like “environmentally-friendly material” or “green material” have been increasingly appearing in papers on material sciences [1,2,3,4,5,6,7,8]. The definitions of such materials change from one author to another and depend upon the grounds used for such terms. On the one hand, there are some papers based on life cycle assessment (LCA) [9,10] that show high technical competence and deliver numerical values on the materials’ environmental impact. However, since LCA involves materials, processes, and application, the same material can deliver different environmental impacts depending on the application or lifespan. On the other hand, some authors base their definition upon the principles of green chemistry [11,12]. These principles focus on the material and on avoiding the use of reagents and the generation of waste. Other studies base the environmental impact of the materials on the renewability of their source. Both LCA and green chemistry principles have their advantages and disadvantages and can deliver opposing conclusions. Here, one can draw conclusions on the environmental impact of the researched materials based upon the principles of green chemistry because the research does not involve application, and because one can consider the impact on the source of the materials to be weak.

In this work, we developed composites made of bleached kraft softwood fibers (BKSF) reinforced with Polylactic Acid (PLA) and evaluated their flexural properties, because we were interested in their use as an alternative to commercially-available materials such as glass fiber (GF)-reinforced polypropylene (PP) composites [6,13,14]. A successful alternative must present similar mechanical properties, lower environmental impacts, and similar or lower costs. This study did not examine the costs since they are highly impacted by economy of scale, and, to the best of our knowledge, the information at hand was insufficient to generate a proper cost analysis. Our interest on PLA-BKSF composites is based on the properties of the individual materials and on the expected properties of their mixtures [15].

PLA is a biopolymer that can be obtained from renewable resources like starch, is biodegradable, and can display better mechanical properties than most polyolefins. Thus, this polymer has attracted the attention of researchers in search of an environmentally friendly alternative to polyolefins [6,16,17,18,19]. Furthermore, its processing temperatures allows the use of lignocellulosic fibers as a reinforcement agent with limited or zero cellulose degradation.

The use of BKSF as the reinforcement agent was based on different factors. First, BKSF is more environmentally friendly than GF. On the one hand, while a huge quantity of energy is needed to obtain GF, BKSF can be obtained from renewable sources or be recovered from used paper [17,20,21]. Through the use of BKSF, processes with a high energy optimization would be used. On the other hand, the fragility of GF hinders its recyclability since the aspect ratio of such fibers decreases noticeably with cycles [22,23]. In some cases, GF has been substituted by natural fibers [9,14,24]. However, since in most cases the matrices were polyolefin-based, the composites were rendered non- biodegradable. This is of importance due to the magnitude of pollution due to non-biodegradable plastics [25,26]. Second, BKSF are bleached. Bleaching processes decrease the presence of lignin in the surface of the fibers, while increasing the presence of cellulose and hemicelluloses [6,17,27,28]. Higher percentages of hemicelluloses were previously correlated with enhanced mechanical properties in the case of polyolefin-based composites, and it was proposed that this was due to the presence of hydroxyl groups on the surface of the fibers that enable the formation of hydrogen bonds with the matrices [22,29]. Nonetheless, the same literature supported the use of coupling agents in making hydrophobic matrices and hydrophilic reinforcements compatible [22]. Coupling agents have been successfully used with lignocellulosic fibers and with GF, the maleic acid- based ones being the most used [30,31,32]. Thus, if a material is made avoiding the use of such reagents, it will agree with the principles of green chemistry [33].

The strength of a composite material is not only highly impacted by the quality of the matrix- reinforcement interface, but also by the intrinsic strength of the phases. In fact, the literature emphasizes how the fiber strength, the fiber/fiber joint strength, the dispersion and mean relative orientation of the fibers, and the number of efficient joints per volume govern the strength of a composite [22,34]. Paper based on softwood shows superior intrinsic strengths compared to hardwood-based ones. In one study, bleached kraft softwood fibers displayed a tensile index of 75.6 Nm/g, while this index was 60.6 Nm/g for bleached kraft hardwood fibers [35]. Another study obtained similar results in terms of network strength [36]. However, there are also studies that show how these intrinsic properties are matrix dependent [8]. This is supported by the different intrinsic properties obtained through direct measurements or through micromechanic models [37]. Moreover, the intrinsic tensile strength of a single fiber can change if measured inside or outside a composite [37].

The measurement of the flexural properties of a composite material allows the evaluation of its anisotropy [3,17,38]. Due to its semi-aligned orientation, mold-injected, short fiber-reinforced composites tend to show different tensile strengths depending on the mean orientation of the fibers relative to the loads. Additionally, flexural conditions are more common than purely tensile ones [9]. Previous studies have proven that strong interfaces can be created in BKSF-reinforced PLA composites without coupling agents. However, to the best of our knowledge, the literature on the flexural strength of such composites is scarce [27,39].

In this paper, we prepared composites based on a PLA matrix reinforced with 15 to 30% w/w of BKSF, tested them under three-point bend conditions, and compared the results with those of uncoupled and coupled GF-reinforced composites. The contribution of the fibers used in the strength of the composite was studied by evaluating the intrinsic flexural strength of the fibers. Other authors use the ratio between the flexural and tensile strengths of the composites instead of the ratio between fiber tensile strength factor (FTSF) and fiber flexural strength factor (FFSF) to obtain the intrinsic flexural strength of the fibers from their intrinsic tensile strength [40]. Nonetheless, in the case of semi-aligned short fiber reinforced composites, the neat contribution of the fibers to the strength of the composite is impacted by the interfacial shear strength, the fiber size of the reinforcements, and its mean orientation, as clearly shown by the modified rule of mixtures, as will be discussed later [40,41]. Furthermore, both experimental values include the contribution of the matrix.

Finally, we compared the contribution of the fibers to the tensile and flexural strengths of the composite, in magnitude and in percentage, to identify similarities and differences.

## 2. Materials and Methods

### 2.1. Materials

Bleached Kraft Softwood fibers (BKSF) derived from *Pinus radiata*, were kindly provided by Celulosa Arauco y Constitución, Santiago, Chile. Polylactic Acid (PLA) under the trade name of Ingeos Biopolymer 3251D of Natureworks was purchased from Resinex, Vilallonga del Camp, Spain. Diethyleneglycol dimethylether (diglyme), with a 162 °C boiling point and a 134,17 g/mol molecular weight used as dispersing agent was provided by Clariant, Malmö, Sweden.

### 2.2. Methods

**Figure 1:**
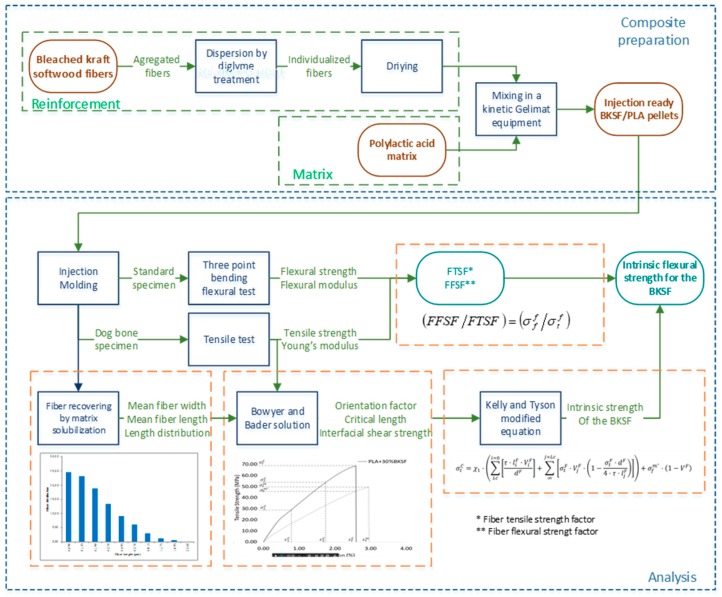
Flowchart of the research, including the preparation and mechanical properties test phases.

Figure 1 presents the flowchart used for the production of composite materials and their characterization.

#### 2.2.1. Preparation of Composite Materials

Prior to its mixing, BKSF were individualized through a disintegration process in a Pulcel pulper equipment (metrotec, Tolosa, Spain). This process was carried out in a 5 wt % suspension of fibers in 2:3 diethyleneglycol dimethylether (diglyme): water. This treatment prevents agglomerations between fibers and favors a good dispersion of the fibers in the composite material. The diglyme interacts via hydrogen bonds with the hydroxyl groups of the fibers and blocks them during the fiber drying process. This interaction enhances the individualization of the fibers during the extrusion process of the material.

The dried fibers were mixed with the PLA matrix in a Gelimat kinetic mixer. The diglyme evaporates at 160 °C out of solution during this process, thus facilitating the interaction between fiber and matrix. The mixer rotates at 300 rpm during the charge of the phases, and then the speed is increased up to 2500 rpm. This speed is maintained for 2 min, when the material reaches a temperature of 195 °C. Once discharged from the mixer the composite was let to cool down and then granulated into pellets using an Agrisma knife mill (Agrisma, Girona, Spain). These pellets were dried at 80 °C for 48 h before processing.

The resulting composite materials were processed by injection molding using an Aurburg 220 M 350–90U equipment (Aurburg, Loßburg, Germany). A temperature ramp of 180, 190, 200, and 210 °C was used in the different zones of the injector, and a first and second pressure of 120 and 37.5 kg·cm^−2^, respectively.

#### 2.2.2. Mechanical Characterization

The characterization of the bending properties was carried out on specimens of UNE-EN ISO 178:2001 standard dimensions. Ten samples of each different material were tested using an Instron 1122 universal testing machine equipped with a 5kN load cell. We used a load element with a radius of 5 mm and with supports located at a distance of 50 mm between them.

The results were analyzed with SPSS Statistics v.25 software (IBM Corp., Armonk, NY, USA) using a one-way analysis of variance (ANOVA) followed by a Tukey mean separation test. We considered differences to be significant for a *P* ≤ 0.05.

#### 2.2.3. Evaluation of the Size Distribution of the Reinforcements

The literature reports that short fiber reinforcement noticeably changes its size distribution during mixing and injection molding processes [15]. Therefore, the reinforcements must be extracted from the composite to evaluate their fiber size distribution. This was done by dissolving the matrix in a Soxhlet apparatus. The used solvent was Decalin. The solvent vapors were refluxed for 24 h. Then the recovered fibers were washed with acetone and water to eliminate any remaining residues. The fibers were dried in an oven at 105 °C for 24 h.

The morphologic analysis of the fibers was conducted in a MorFi Compact from Techpap SAS, Valence, France. The analysis includes mean fiber length and width, fiber length and diameter distributions, as well as fines content. Four batches of each type were performed, in agreement with ISO/FDIS 160652.

## 3. Results and Discussion

### 3.1. Flexural Strength Properties of PLA–BKSF Composites

We prepared composites made of PLA with different amounts of BKSF, ranging from 15% to 30%, injection-molded them into test specimens, and characterized their flexural properties using a bend strength test (Figure 1). The results of the bending test are summarized in Table 1, where *σ_f_^C^* is the flexural strength, σ_f_^m^* is the flexural strength of the matrix at the maximum composite strength, *D* is the experimental deflection of the studied materials during the bending test, and *ε_f_^C^* is the strain of composites at the maximum flexural strength value. The *ε_f_^C^* was calculated from the formula *ε_f_^C^* = *(6·D·d)/L^2^*, where D is deflection, *d* is the width of the bending specimens, and *L* is the length of the support span. The σ_f_^m^* value was calculated from the fit of the experimental stress-deflection curve.

**Figure 2:**
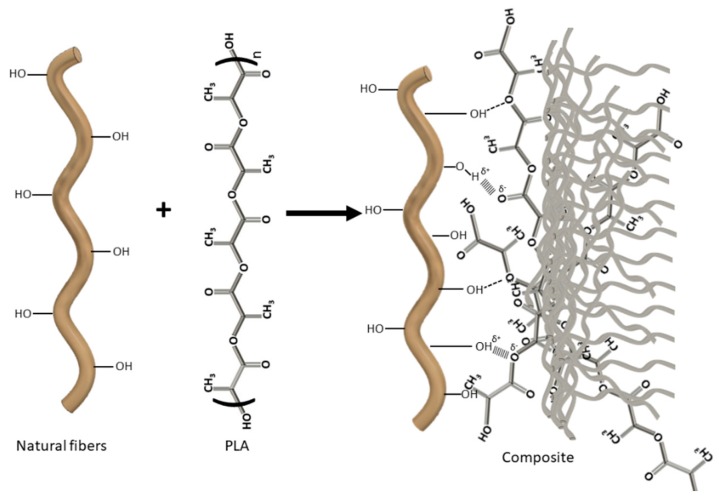
Theoretical scheme showing the potential interactions between BKSF and PLA.

We observed that the flexural strength of the composite materials increased linearly with a coefficient of determination (r^2^) of 0.983. Generally, this lineal correlation is indicative of a homogeneous dispersion of the fibers in the polymer matrix and an adequate interface. The differences in deformation resistance between the different composites were statistically significant (*P* ≤ 0.05), as determined by ANOVA analysis. However, no significant increases were observed in composites with higher BKSF content than 30%, probably due to less homogeneous fiber dispersion. The flexural strength of composite materials is affected by the properties of the matrix and of the reinforcement, by their size distribution, their intrinsic tensile strength, by the orientation and volume of the reinforcement in the material, and most importantly, by the interactions established between both phases or the interfacial shear strength (IFSS). This is because of the stress transfers from the matrix to the reinforcement. We believe that the relatively good interaction between the BKSF and the PLA matrix is due to the formation of hydrogen bonds and Van der Waals interactions, and later in the paper, we computed the strength of the interface (Figure 2).

As it is known, bleached fibers have only aliphatic hydroxyl groups on their surface, unlike mechanical pulp fibers. As has been shown in previous works, the presence of these aliphatic hydroxyl groups allows a greater interaction with the polymer chains [42].

We observed a maximum increment of 43.8% in the flexural strength for the 30% BKSF composite material compared to PLA alone. This value is considerably lower than that obtained from other natural fiber-reinforced composites using polymer matrices such as polyethylene (PE), polypropylene (PP), polyamide 11 (PA11), and starch [33,43,44,45,46]. However, the nominal flexural strength of PLA–BKSF composites at the same fiber percentage is only surpassed by starch–fiber composites [45]. Probably due to the greater ability of the fibers to interact with thermoplastic starch as opposed to PLA which has a lower capacity. Nonetheless, the flexural strength of the PLA matrix is 60 MPa. This is around 20 MPa higher than that of PP, PA11, and starch, and much higher than that of PE.

On the other hand, the deflection of the composite materials was only slightly affected by the addition of the cellulosic BKSF. A maximum reduction of 0.26% was observed for the composite with 30% of BKSF. Generally, the deflection value of the polymeric matrix alone is drastically reduced in composite materials by the addition of a stiffer phase such as cellulosic fibers. Nonetheless, the PLA matrix has a low deformation profile and this might have reduced the effect of fiber addition in the material.

**Figure 3:**
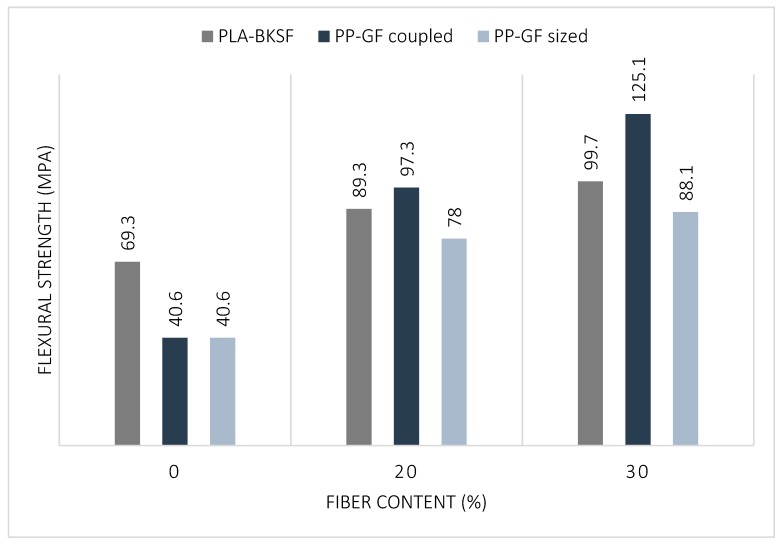
Comparison between PLA–BKSF and PP–GF composites.

One of the objectives of the production of fully bio-based composite materials is the replacement of common composites that are typically produced with petrol-based matrices and synthetic reinforcements. The most successful examples of such materials are GF-reinforced, PP composites. Hence, we compared the flexural strength results obtained for PLA–BKSF composites with those of PP–GF composites at different fiber content percentages (Figure 3). Two different types of PP–GF composites were used for comparison: one that contained a coupling agent in the formulation (PP–GF coupled), and another one where the GF’s surface was modified in order to decrease the polarity of the fibers and promote a correct dispersion in the composite material (PP–GF sized).

The results showed that the PLA matrix alone was stronger than the PP matrix alone. Also, PLA–BKSF composites had higher flexural strengths than PP–GF sized, but not PP–GF coupled, at the same fiber content. It should be noted that with the compound PLA and 30% BKSF it is possible to achieve 80% of the flexural strength of a PP composite with 30% glass fiber coupled and to exceed the strength of 30% sized glass fiber. However, it should be noted that by means of a PLA compound and 30% BKSF it is possible to replace a PP compound with a 20% glass fiber coupled. From these results, GF seem to have a better reinforcing effect than BKSF, and we believe this is due to the stronger mechanical properties of GF compared to cellulosic fibers. However, the higher flexural strength of PP–GF coupled could also be the result of a better interface given by the use of a coupling agent. Moreover, BSKF are less dense (around 1.5 g·cm^−3^) than GF (2.5 g·cm^−3^) [47,48] and they clearly reduce the increment in the composite materials’ density at increasing percentages of reinforcement content within the polymer matrix.

### 3.2. Intrinsic Flexural Strength Properties

In order to evaluate the FTSF, the mechanical properties of the PLA + 30% BKSF composite were solved using the widely reported modified rule of mixtures for the modelling of the tensile properties of materials (Equation (1)) [49,50].

(1) $$\sigma_{t}^{C}=f_{c}\cdot \sigma_{t}^{f}\cdot V^{F}+\sigma_{t}^{m^{*}}\cdot \left(1-V^{F}\right)$$

The tensile strength of the compound ($\sigma_{t}^{C}$) is obtained by multiplying a coupling factor ($f_{c}$) by the intrinsic tensile strength of the fiber ($\sigma_{t}^{f}$) and by the volume fraction of the reinforcement ($V^{F}$) followed by the addition of the tensile strength of the matrix at the point of failure ($\sigma_{t}^{m^{*}}$) multiplied by the fraction in volume of the matrix. With this, we can determine the contribution of the matrix and the reinforcement to the properties of the compound, and also a coupling factor that represents the effectiveness of the fibers as a reinforcement. In fact, *f_c_* can be expressed as *f_c_* = *χ*_1_·*χ*_2_, where *χ_1_* is an orientation factor that accounts for the mean orientation of the fibers against the loads, and *χ*_2_ is a length and interface factor that accounts for the impact of the size distribution of the reinforcements and the interfacial shear strength and the intrinsic tensile strength of the fibers [42,51]. The previously obtained values of tensile properties for 15–30 wt % of BKSF were used in the study of the tensile micromechanical properties [39]. A tensile strength of 65.3 MPa was determined for the composite with 30% BKSF, as well as a Young’s modulus of 6.19 GPa with a deformation at break of 2.2%.

Previous works have reported a modified rule of mixtures for the study of bending properties (Equation (2)).

(2) $$\sigma_{f}^{C}=f_{c}^{f}\cdot \sigma_{f}^{f}\cdot V^{F}+\sigma_{f}^{m^{*}}\cdot \left(1-V^{F}\right)$$

where $\sigma_{f}^{C}$, $\sigma_{f}^{f}$, and $\sigma_{f}^{m^{*}}$ are the flexural strength of the composite, reinforcement, and matrix, respectively. The efficiency factors are represented as $f_{c}^{f}$, and they include the effect of the interface, the orientation of the fibers, and their aspect ratio (l/d) inside the material.

In both cases, the equation contains two incognita because it is not possible to measure the intrinsic tensile strength of the fibers. However, the product of $f_{c}\cdot \sigma_{t}^{f}$ or $f_{c}^{f}\cdot \sigma_{f}^{f}$ corresponds to the fiber flexural strength factor (FFSF), which is an indicator of the effect of the reinforcing fibers in the composite [41].

**Figure 4:**
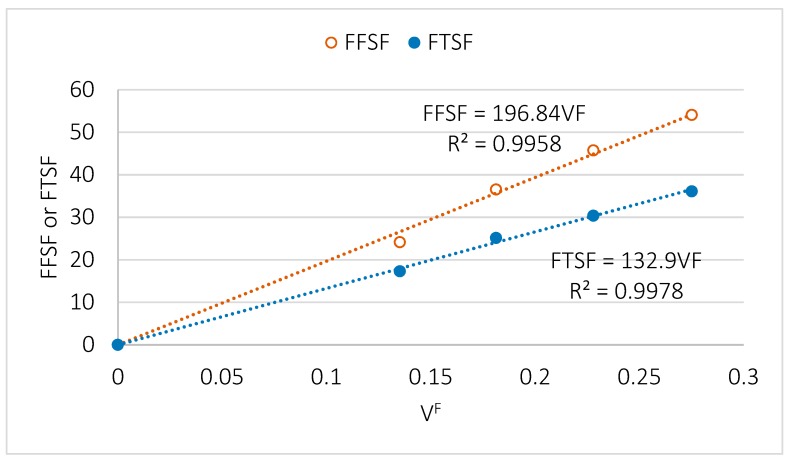
FFSF and FTSF of PLA-BKSF composites. The VF value is the volume fraction of fibers in the composite.

We calculated the FFSF for PLA–BKSF by performing a lineal regression analysis using all the studied composites and obtained a value of 196.84 MPa (Figure 4).

Instead, by calculating the fiber flexural strength factor (FFSF) and fiber tensile strength factor (FTSF) (Equation (3)) it is possible to calculate the value of the neat contribution of the BKSF to the flexural strength of the composites without this being influenced by the contribution of the matrix.

(3) $$FFSF=f_{c,f}\cdot \sigma_{f}^{f}=\left(\frac{\sigma_{f}^{c}+\sigma_{f}^{m^{*}}\cdot \left(1-V^{F}\right)}{V^{F}}\right);\;FTSF=f_{c}\cdot \sigma_{t}^{f}=\left(\frac{\sigma_{t}^{c}+\sigma_{t}^{m^{*}}\cdot \left(1-V^{F}\right)}{V^{F}}\right)$$

We also calculated the intrinsic tensile strength of the reinforcement (*σ_t_^f^*) by using the Kelly and Tyson modified equation and its solution, provided by Bowyer and Bader [52,53]. With this, we then obtained the theoretical intrinsic flexural strength value of BKSF (*σ_f_^f^*) by using the ratio between FFSF and a fiber tensile strength factor (FTSF):

(4) $$\sigma_{f}^{f}=\frac{FFSF}{FTSF}\cdot \sigma_{t}^{f}$$

The results obtained for FTSF and FFSF for the different percentages of reinforcement are represented in Figure 4. This value is similar to the ones previously obtained with other natural fiber- reinforced composites [41,43,45], and superior to the ones obtained with the PA11-natural fibers composites [33]. In PA11-based composites, the lower reinforcement effect provided by the natural fibers was thought to be derived by the lower quality of the interface, which was formed by H-bonds instead of covalent bonds such as those formed in PP-based composites. A similar result was expected in our PLA–BKSF composites since the interface is also obtained by intermolecular forces. Surprisingly, we found a higher value (132.9) for the tensile analogous factor of the composite materials, the FTSF (Fiber Tensile Strength Factor), than that previously observed for pine fiber-reinforced, polymer matrix composites [33,43]. Nonetheless, this value is within the range of other natural fibers [9,15,45] (Figure 4).

**Figure 5:**
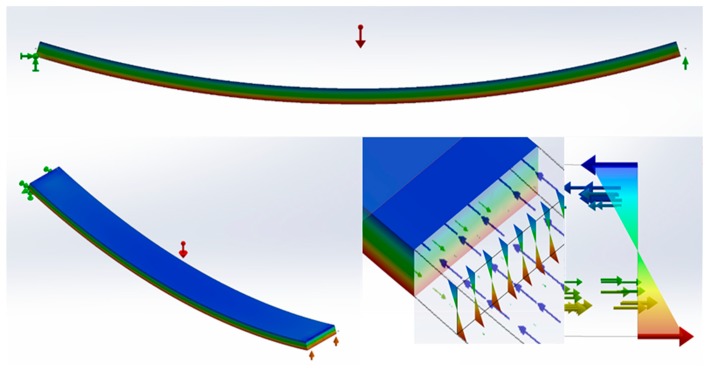
Scheme of tensile and compression forces combination during the flexural test. In the color range green to red the tensile forces and in the color range blue the compressive forces.

Flexural strength tends to be higher in value than tensile strength, due to the combination of tensile and compression forces during the bending test. The combination of both forces also implies a higher contribution of the fibers to the flexural strength (Figure 5).

Some authors link the difference with the anisotropy of semi aligned, short fiber-reinforced composites and the loads at the section of the specimens [33]. While tensile specimen sections are fully loaded under tensile forces, flexural specimens are loaded under compression and tensile forces. Most polyolefins have higher compressive strengths than tensile strengths, and therefore, the portion of the specimen section under compression is expected to contribute more than the portion under tensile to the flexural strength of the composite.

A higher FFSF compared to FTSF is usually expected. However, their ratio in PLA–BKSF composites (1.48) is small when compared to that of other natural fiber-reinforced composites where the value is around 1.7 [41,54], and similar to that of GF-reinforced composites [41]. Nevertheless, the value of this ratio is in the range of those expected for composite materials reinforced with lignocellulosic fibers.

The calculation of the intrinsic properties of natural fibers is quite difficult and expensive. However, it is possible to back-calculate the intrinsic strength of a fiber by using lineal models such as mRoM [55]. The value of *σ_f_^f^* can be derived from *σ_f_^f^* = (*FFSF*/*FTSF*)·*σ_t_^f^* [43]. Nonetheless, the value of *σ_t_^f^* in our composites is unknown. The literature provides a value of *σ_t_^f^* of a bleached softwood fiber in the range of 800–900 MPa [42]. The matrix, the equipment, and the process to obtain the composite materials have a considerable impact in the mechanical properties of the fibers such as *σ_t_^f^*. The *σ_t_^f^* of BKSF was calculated using the Bowyer–Bader solution from the Kelly–Tyson equation [52,53,56], as reported in previous papers [13,29,57,58].

**Figure 6:**
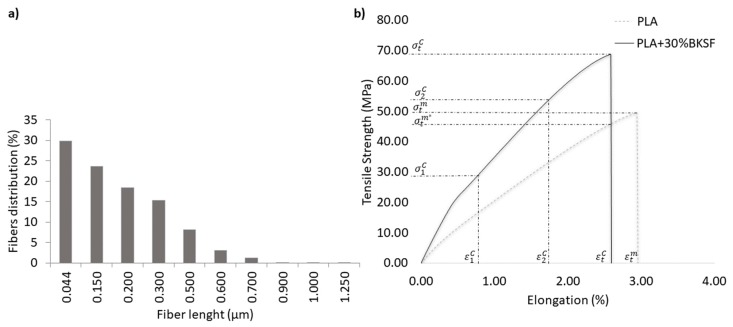
(**a**) Fiber lengths distribution of PLA + 30BKSF. (**b**) Elongation-tensile strength curves of the PLA matrix and the composite reinforced with 30% BKSF.

Figure 6 shows the fiber size distribution of the BKSF extracted from the composite containing 30 wt % of reinforcement and the experimental data used to solve the modified Kelly and Tyson equation.

Kelly and Tyson proposed a modification of an mRoM (Equation (5)), where the *f*_C_ is disintegrated in the orientation factor (*χ*_1_) and the contribution of the subcritical and supercritical fibers and the interfacial shear strength (*τ*) *l^F^* is the fiber length and *d^F^* the fiber diameter:

(5) $$\sigma_{t}^{C}=\chi_{1}\cdot \left(\overset{i=0}{\underset{Lc}{\sum }}\left[\frac{\tau \cdot l_{i}^{F}\cdot V_{i}^{F}}{d^{F}}\right]+\overset{j=Lc}{\underset{\infty }{\sum }}\left[\sigma_{t}^{F}\cdot V_{j}^{F}\cdot \left(1-\frac{\sigma_{t}^{F}\cdot d^{F}}{4\cdot \tau \cdot l_{j}^{F}}\right)\right]\right)+\sigma_{f}^{m^{*}}\cdot \left(1-V^{F}\right)$$

In order to determine the contribution of subcritical and supercritical fibers it is necessary to calculate the value of the critical length. The critical length is determined according to the shear-lag model that allows the distribution of efforts in the reinforcement to be determined. According to this model, the polymer matrix transmits the stresses applied to the material on the interface by shear forces. Consequently, the fibers will have a zero load at their ends and a full load at the center of their length. Therefore, depending on the length of each fiber inside the composite, the load in its center will be higher or lower than its intrinsic tensile strength. Thus, the critical length will be the length of the fiber where in the center of its length the load is equal to the intrinsic tensile strength. The critical length of the fibers is determined by the outer area of the fibers and the ability to transmit the stresses from the matrix to the reinforcement (quality of the fiber-matrix interface). The critical length being equal to the product of fiber radius and intrinsic tensile strength of the fibers, divided by the interfacial shear strength. The fibers with a length greater than the critical length are referred to as supercritical and the fibers with a length less than the critical length are referred to as subcritical.

Applying the solution proposed in the Bowyer and Bader model to the results obtained for the 30% BKSF compound, the orientation factor (*χ*_1_ = 0.29), the interfacial shear strength (*τ* = 28.17) close to the value of the Von misses ($\sigma_{t}^{C}/\sqrt{3}$) criteria (28.7) and the critical length (*L*_c_ = 368.11 μm) were determined. These values allowed the calculation of the intrinsic resistance of the fibers (*σ_t_^f^*) for the different composites with an average value of 769.42 MPa. This result is slightly lower than that observed in the literature (800–900 MPa) [59,60,61,62]. In order to corroborate the model used, the theoretical tensile strength of the different composite materials was calculated using the Kelly–Tyson equation and the modified rule of mixtures (Table 2).

As shown in Table 2, there is a strong correlation between the tensile strength results of composites and the values obtained by micromechanical resolution. By applying the previously calculated factor of 1.48 to the fibers’ intrinsic tensile strength, we obtained a value of fiber intrinsic flexural strength of 1126.75 MPa. Furthermore, by using macroscopic properties, a slightly lower value is obtained for the intrinsic flexural strength of the fibers (1104 MPa).

The dispersion of the fibers in the matrix is another factor that must be considered. It is well known that bleached fibers are difficult to disperse through a non-polar matrix because they are highly polar. In our studies, a dispersant agent was necessary to obtain the composite material and even then, the dispersion was not totally adequate. We present the coupling factors obtained by the resolution of the respective modified rules of mixtures (Equations (2) and (3)) of the bending and tensile tests in Table 3. The coupling factor can be broken down into two factors: the length factor or l/d, and the interface quality. In bleached kraft softwood fibers, the main coupling factor is the interface of the material. Our results show coupling factors close to 0.17, slightly lower than the value of *f*c = 0.2 [51], which is accepted for well-bonded systems. It is well described in the literature that PLA-natural fiber composites do not obtain a totally well-bonded system due to the poor energetically-favored interactions between PLA and cellulosic materials [63,64,65,66]. With the exception of 15% composites, the values obtained for $f_{c}$ and $f_{c}^{f}$ are similar. These results allow us to affirm that the orientation factor and the length and interface factor are similar for the tensile and bending properties.

The Bowyer–Bader solution proposed to estimate the *σ_t_^f^* from *σ_t_^f^* = *E_t_^f^*·*ε_t_^c^*, where *E_t_^f^* is the intrinsic tensile modulus of the fiber and *ε_t_^c^* is the tensile elongation of the composite materials. Afterwards, the Kelly–Tyson equation can be expressed as:

(6) $$\sigma_{t}^{C}=\chi_{1}\left(X+Y\right)+Z$$

where *X*, *Y*, and *Z*, are the contributions of the subcritical length fibers, supercritical length fibers, and the matrix, respectively. Then, using two different strain levels and its maximum tensile strength at such a strain level it is possible to estimate each contribution (Figure 6b):

(7) $$R=\frac{\sigma_{t1}^{C}-Z_{1}}{\sigma_{t2}^{C}-Z_{2}};\;R^{*}=\frac{\left(X_{1}+Y_{1}\right)}{\left(X_{2}+Y_{2}\right)}$$

**Figure 7:**
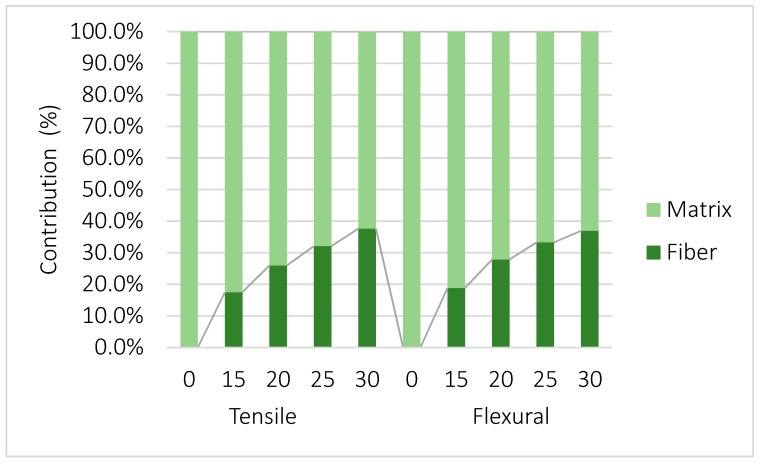
Contributions of the matrix and the reinforcements to the tensile (**left**) and flexural (**right**) strengths of the composites at different fiber content percentages (x axis).

The nominal contributions of the fibers and the matrix are noticeably higher in the case of the flexural strength. The higher contributions of the fibers can be explained by the higher strains measured under flexural loads before breaking. However, the percentage contribution of both phases is similar (Figure 7). Due to their aspect ratios, the reinforcements are expected to work very well under tensile forces but to be prone to buckling under compression.

The contributions of the fibers are very similar in both cases in shape and in values, all within a 2% range and without statistically significant differences. Thus, both phases contribute similarly to the tensile and flexural strengths, and the main cause of a higher flexural strength can be attributed to the compressive strength of the matrix.

## 4. Conclusions

In this work, flexural strength of fully biodegradable composite materials of PLA and BKSF were analyzed. Our macromechanical results show an increase in the flexural strength of the materials with higher fiber content. At the same time, the deformation of the materials was only slightly reduced by the increasing fiber content due to the high stiffness of the PLA matrix. Our results show that PLA–BKSF composites are suitable to replace PP–GF sized composites with the same fiber content. However, the highest flexural strength was obtained with the PP + 30% GF composite. The density of PLA–BKSF composites is considerable higher than PP–GF composites at the same fiber content due to the higher density of PLA compared to PP. Although it should be noted that with the composite PLA + 30% BKSF it is possible to replace PP + 20% GF coupled, obtaining a material with the same mechanical properties from renewable resources.

Our micromechanical analysis shows that the FFSF is higher than the FTSF in PLA–BKSF composites, as expected. However, the FFSF has a similar value in other pine fiber-reinforced composite materials, while the FTSF is quite high. By studying the micromechanics of the flexural strength of PLA–BKSF composites, we determined the intrinsic flexural strength of the fibers as well as their coupling factor.

## Figures and Tables

**Table 1:** Flexural strength (*σ_f_*^C^), deflection (D) and strain (*ε_f_*^C^) of the polylactic acid with bleached kraft softwood fibers composites (PLA–BKSF). The contribution of the matrix (*σ_f_*^m^*) has also been included.

Samples	*V*_f_	*σ_f_*^C^ (MPa)	*D* (mm)	*ε_f_*^C^ (%)	*σ_f_*^m^* (MPa)
PLA	0	69.3 ± 0.9	3.4 ± 0.3	2.26	69.3
PLA + 15% BKSF	0.135	81.1 ± 1.0	3.2 ± 0.1	2.12	65.8
PLA + 20% BKSF	0.181	89.3 ± 0.7	3.1 ± 0.4	2.06	64.4
PLA + 25% BKSF	0.228	95.5 ± 1.1	3.1 ± 0.4	2.06	64.4
PLA + 30% BKSF	0.275	99.7 ± 0.5	3.0 ± 0.2	2.00	62.9

**Table 2:** Comparison between experimental and back calculated tensile strengths of the composite materials.

Fiber Content (%)	*σ_t_^f^* (MPa)	*σ_t_^c^*_exp_ (MPa)	*σ_t_^c^*_KT_ (MPa)	*σ_t_^c^*_RofM_ (MPa)
15	705.59	57.5	58.33	58.09
20	820.39	62.9	62.09	61.78
25	772.06	65.6	65.55	65.43
30	779.64	68.8	68.56	69.15
	769.42		*R*^2^ = 0.985	*R*^2^ = 0.975

**Table 3:** Coupling factor (*f*_c_) of PLA–BSKF composites by flexural and tensile properties.

Fiber Content (%)	*V* ^F^	*σ_f_^f^* (MPa)	*f*_c_^f^	*f*_c_^t^
15	0.135	984	0.182	0.155
20	0.181	1192	0.169	0.175
25	0.228	1163	0.173	0.174
30	0.275	1168	0.168	0.170
